# Case of widespread and atypical cutaneous leishmaniasis in a young woman with breast cancer

**DOI:** 10.1002/ccr3.9345

**Published:** 2024-08-19

**Authors:** Fatemeh Mohaghegh, Fatemeh Mokhtari, Fereshteh Sherafat, Mohammad Shoushtarizadeh, Elham Tavousi Tabatabaei

**Affiliations:** ^1^ Department of Dermatology, Skin diseases and Leishmaniasis Research center, School of Medicine Isfahan University of Medical Sciences Isfahan Iran; ^2^ Department of dermatology University of Pittsburgh school of medicine Pittsburgh Pennsylvania USA

**Keywords:** cutaneous leishmaniasis, breast cancer, leishmaniasis, malignancy

## Abstract

Cutaneous leishmaniasis is caused by protozoan parasites of the genus leishmania. Atypical presentation and widespread progression of the lesions may be seen in an immunocompromised patient. We report a case of atypical and widespread cutaneous leishmaniasis in a young woman with breast cancer.

## 1 INTRODUCTION

Leishmaniasis, a vector‐borne disease caused by protozoa of the genus Leishmania, is endemic in Iran according to the World Health Organization, with most cases attributed to L. major and L. tropica species.^1^, ^2^, ^3^ The disease presents a spectrum of cutaneous manifestations, influenced by factors such as parasite virulence, host immune response, age, nutritional status, and inoculation site. Immunocompromised patients generally exhibit a higher likelihood of atypical clinical presentations of the cutaneous disease.^4^

The development of leishmaniasis has been reported in patients with various types of malignancies, including solid tumors such as colorectal cancer and head and neck carcinoma, as well as hematological malignancies like leukemia and lymphoma. Cases of leishmaniasis co‐occurring with skin cancers, including basal cell carcinoma and squamous cell carcinoma, have also been documented.^5^, ^6^

However, to our knowledge, there have been no reported cases of widespread atypical Cutaneous Leishmaniasis characterized by multiple cutaneous nodules in a breast cancer patient following chemotherapy.

## 2 CASE HISTORY

In November 2021, a 26‐year‐old female with a history of invasive ductal carcinoma diagnosed in 2019 was referred for evaluation of multiple painful skin plaques and papules. The patient reported the initial appearance of a single vesicular lesion on her elbow 3 months prior, which subsequently progressed to involve her right upper extremities, back, and the area above her right knee. She denied any history of prolonged fever, nasal congestion, or glove and stocking hypoesthesia. The patient had undergone chemotherapy and radiation therapy for breast cancer. The cutaneous lesions developed during her chemotherapy regimen with Exemestane, at a time when there were no signs of active breast cancer. Prior to referral, the lesions had been unsuccessfully treated as herpes zoster with antiviral therapy.

**FIGURE 1:**
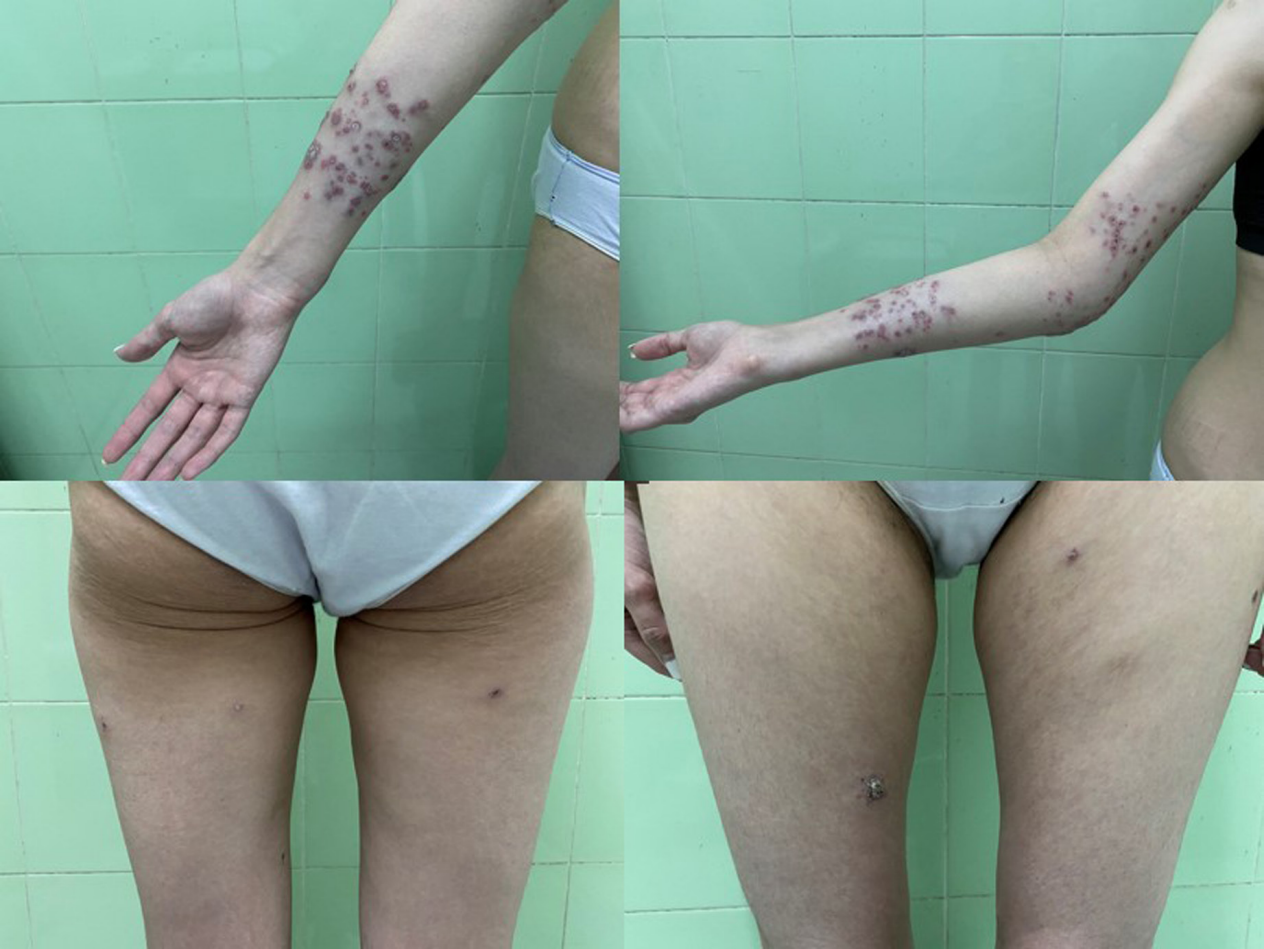
Multiple tender, erythematous papules and plaques most prominently around elbows, forearms and right thigh.

**FIGURE 2:**
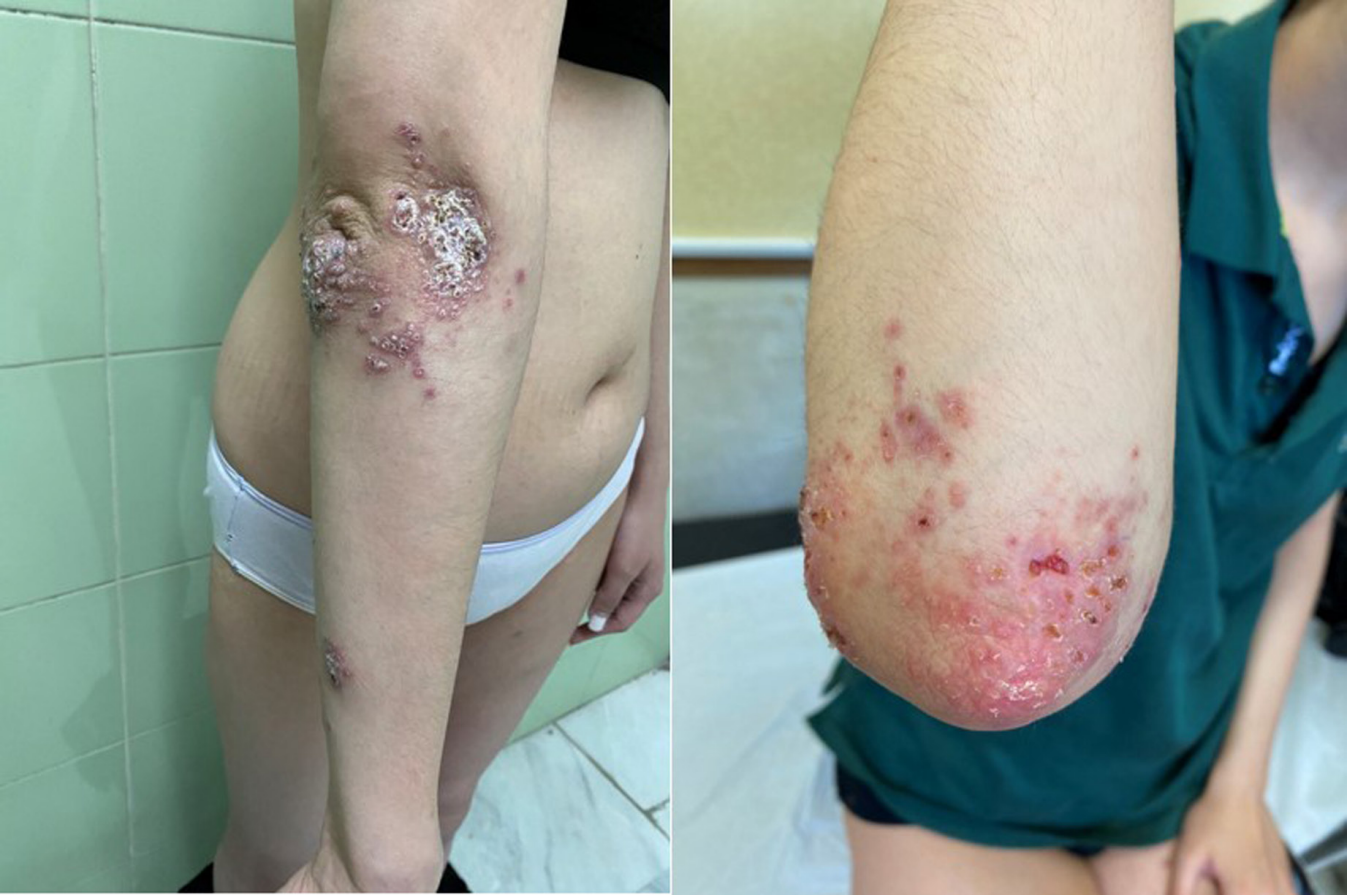
The plaques in some areas were inflamed and ulcerated and some were erythematous with crusts.

Full‐body cutaneous examination revealed multiple tender, erythematous papules and plaques, most prominent around the elbows, forearms, and right thigh (Figure 1). Some lesions around the elbow had enlarged to form crusted or verrucous plaques. In certain areas, the plaques were inflamed and ulcerated, while others were erythematous with crusts (Figure 2). The patient also presented with painful subcutaneous nodules on the extremities and sporotrichoid lesion patterns.

## 3 METHOD (DIFFERENTIAL DIAGNOSIS, INVESTIGATIONS, AND TREATMENT)

Given the widespread papules and plaques with ulcerations and crusts, subcutaneous nodules, sporotrichoid lesions, and the patient's residence in a Leishmaniasis‐endemic area, the differential diagnoses included sporotrichosis, leishmaniasis, and psoriasis. Systemic examination revealed no other notable findings.

A skin biopsy from the upper extremity showed superficial and deep perivascular infiltration of histiocytes, plasma cells, and lymphocytes. No evidence of special organisms or suppurative granulomas characteristic of sporotrichosis was found, and Leishman bodies were not observed. However, the specimen was reported as highly suggestive of an infectious process.

**FIGURE 3:**
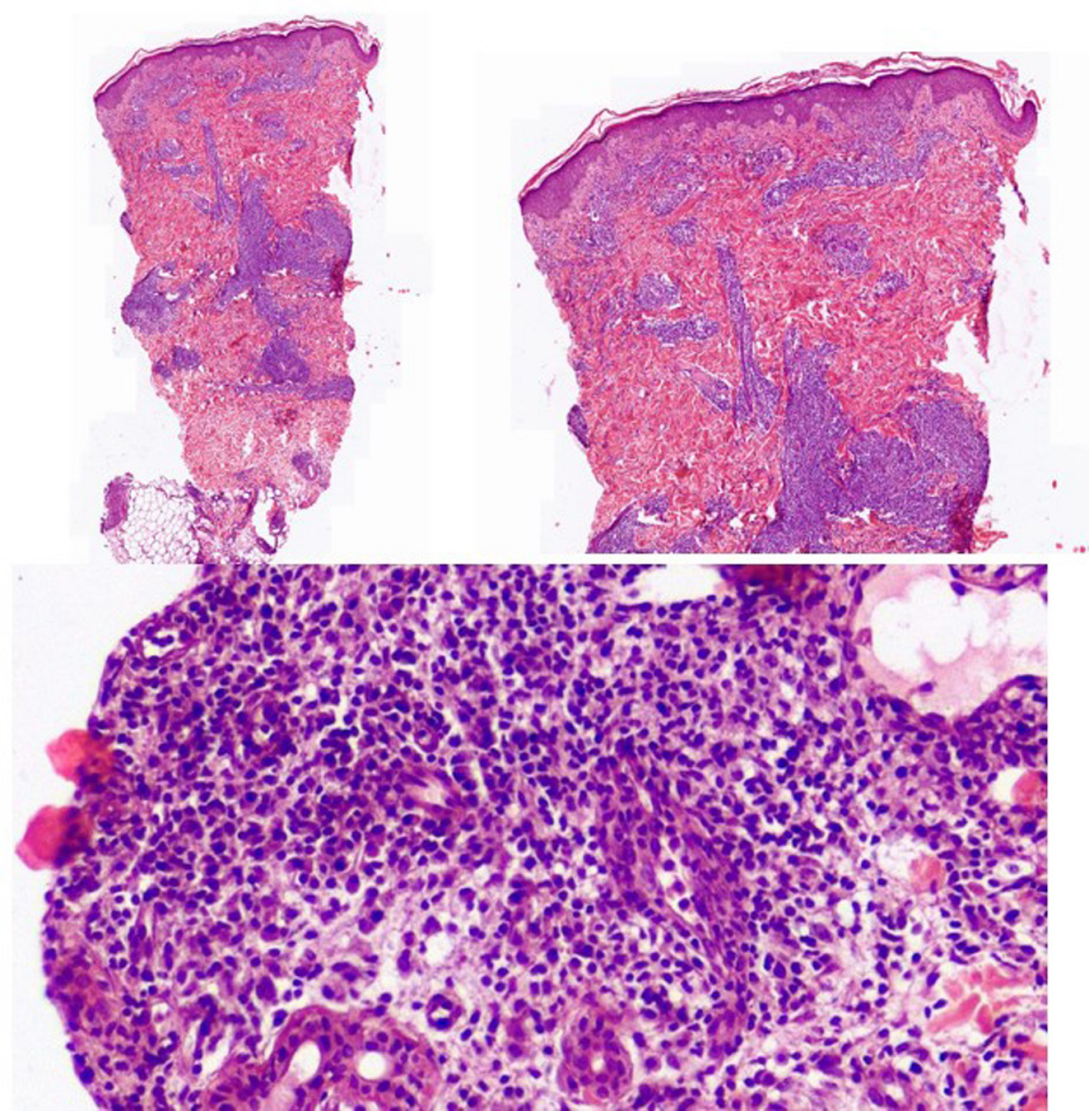
Giemsa staining, revealing a few Leishmania amastigotes.

Direct skin smears from four lesions in different cutaneous areas were collected and examined via Giemsa staining, revealing a few Leishmania amastigotes (Figure 3).

Following diagnosis confirmation, the patient was initiated on anti‐protozoan treatment with intramuscular meglumine antimoniate (glucantime) at 20 mg/kg/day for 21 days. After a three‐week interruption, treatment was resumed at a lower dosage for an additional 21 days. Initial therapy was moderately successful, with clearance of over 70% of lesions; all subcutaneous nodules and sporotrichoid lesions appeared to have resolved.

## 4 OUTCOME AND FOLLOW‐UP

On follow‐up examination, a few lesions remained active. Despite positive smear results, the patient declined a third treatment course.

## 5 DISCUSSION

Leishmania is a genus of protozoa with over 20 species capable of causing vector‐borne disease in humans.^7^ Transmitted by sandflies of the Phlebotomus and Sergentomyia genera, it presents as three main clinical syndromes: cutaneous, mucosal, and visceral leishmaniasis. Cutaneous leishmaniasis is the most prevalent form of the disease globally. In Iran, anthroponotic cutaneous leishmaniasis (ACL) and zoonotic cutaneous leishmaniasis (ZCL), caused by *L*. tropica and L. major respectively, are the most common forms.^8^

The clinical presentation of Leishmania infection varies greatly with the host's immune status. Intracellular Leishmania infection is controlled by TH‐1 mediated immune response, and patients with defective immune responses are at higher risk for more severe and atypical forms of infection.^9^ Diffuse cutaneous leishmaniasis (DCL) is a rare form associated with immune‐deficiency, caused mainly by L. aethiopica, L. mexicana, and L. amazonensis. DCL is characterized by nodules and plaques that do not ulcerate and very slowly spread to distant locations, commonly affecting the face and extensor limb surfaces.^10^, ^11^ Notably, L. major has been shown to be involved in cases of DCL in acquired immunodeficiency syndrome (AIDS) patients.^12^

There is growing evidence of a potential association between leishmaniasis and malignancy.^5^, ^6^, ^13^, ^14^ Leishmaniasis can mimic malignant lesions, complicating diagnosis, and cases of co‐existing Leishmaniasis and cancer have been reported. Studies have found malignancies, predominantly skin cancers, developing at sites of previous leishmanial lesions or scars. Conversely, leishmaniasis has been observed as an opportunistic infection in patients with existing malignancies, particularly those with hematological cancers.^5^, ^13^

In this context, we present a unique case of leishmaniasis in a breast cancer patient. Our patient, undergoing chemotherapy for breast cancer, presented with an unusual, widespread cutaneous leishmaniasis. The disease atypically ulcerated and progressed rapidly through the extremities, sparing the face and neck, and was accompanied by cutaneous nodules. This atypical presentation led to a relatively late diagnosis. Initial biopsy results were inconclusive, with no amastigotes or Leishman bodies apparent in the specimen. The patient resided in an area endemic for L. major, which provided the only initial diagnostic clue, later verified by smear results. The history of insect bite was unclear.

This case highlights the importance of considering leishmaniasis in the differential diagnosis for immunocompromised patients presenting with atypical lesions in endemic areas. Based on our experience, we recommend that Leishmania direct skin smear testing from multiple lesions be performed earlier in the diagnostic course for such patients, especially when initial biopsy results are inconclusive.

Atypical manifestations of leishmaniasis often lead to delayed diagnosis, putting immunocompromised patients at higher risk for complicated disease and misdiagnosis.^15^, ^16^, ^17^, ^18^, ^19^ In immunosuppressed individuals, particularly those with severe immunosuppression, cutaneous leishmaniasis may exhibit diverse clinical manifestations with atypical and often more severe forms, potentially including visceral involvement.^20^

The development of leishmaniasis in our breast cancer patient raises intriguing questions about potential interactions between these conditions. The occurrence of cutaneous leishmaniasis may be primarily attributed to the immunosuppressive effects of chemotherapy, increasing susceptibility to opportunistic infections. The atypical presentation and extensive spread of cutaneous lesions could be a secondary consequence of this altered immune state.^21^ Additionally, the immunomodulatory effects of breast cancer itself may have played a role in altering the typical disease course. This bi‐directional relationship has been observed in other cases, particularly with hematological cancers, where leishmaniasis has developed as an opportunistic infection.^5^, ^13^

Recent research has explored the deliberate use of Leishmania parasites in cancer therapy. Caner et al. demonstrated that live‐attenuated Leishmania strains, particularly L. tropica, could induce a strong anti‐tumor immune response in a breast cancer mouse model. Although our case represents an inverse scenario, it underscores the complex immunological relationship between these conditions.^21^

While definitive conclusions cannot be drawn from a single case, this report highlights the need for vigilant monitoring of opportunistic infections in cancer patients, particularly those undergoing immunosuppressive treatments. The significance of this case lies in it being the first reported instance of leishmaniasis in a breast cancer patient, opening new avenues for research into the interaction between these two conditions. Further investigation is needed to explore potential relationships between leishmaniasis and breast cancer, which could lead to improved diagnostic and therapeutic strategies for both conditions in the future.

## AUTHOR CONTRIBUTIONS

**Fatemeh Mohaghegh:** Conceptualization; data curation; project administration; resources; validation; visualization; writing – original draft. **Fatemeh Mokhtari:** Visualization; writing – original draft. **Fereshteh Sherafat:** Writing – original draft; writing – review and editing. **Mohammad Shoushtarizadeh:** Writing – review and editing. **Elham Tavousi Tabatabaei:** Writing – review and editing.

## FUNDING INFORMATION

The authors received no financial support for this study.

## CONFLICT OF INTEREST STATEMENT

The authors deny any kind of conflict of interest.

## ETHICS STATEMENT

This study has obtained ethical approval from the Isfahan University of Medical Sciences.

## CONSENT

Written informed consent was obtained from the patient to publish this report in accordance with the journal's patient consent policy.

## Data Availability

All data used and analyzed during this study are available from the corresponding author upon reasonable request.
